# Supplementary material for: Use of Indocyanine Green (ICG), a Medical Near Infrared Dye, for Enhanced Fluorescent Imaging—Comparison of Organic Anion Transporting Polypeptide 1B3 (OATP1B3) and Sodium-Taurocholate Cotransporting Polypeptide (NTCP) Reporter Genes
Source: Molecules. 2019 Jun 21;24(12):2295. doi: 10.3390/molecules24122295 (PMC6630416; doi:10.3390/molecules24122295)
Supplement: Supplementary file 1 [file molecules-24-02295-s001.pdf]

Supplementary data

For

Use of Indocyanine green (ICG), a medical near infrared dye, for enhanced fluorescent imaging application — Comparison of organic anion transporting polypeptide 1B3 (OATP1B3) and sodium-taurocholate cotransporting polypeptide (NTCP) reporter genes

Menq-Rong Wu [1,2], Yi-You Huang [1], and Jong-Kai Hsiao [2,3,*]

[1] Institute of Biomedical Engineering, National Taiwan University, No. 1, Sec. 4, Roosevelt Rd., Taipei 10617, Taiwan

[2] Department of Medical Imaging, Taipei TzuChi General Hospital, Buddhist Tzu-Chi Medical Foundation, No.289, Jianguo Rd., Xindian Dist., New Taipei city 23142, Taiwan

[3] School of Medicine, Tzu Chi University, No.701, Sec. 3, Zhongyang Rd. Hualien 97004, Taiwan

* Correspondence: jongkai@tzuchi.com.tw; Tel.: +886-2-6628-9779-61114

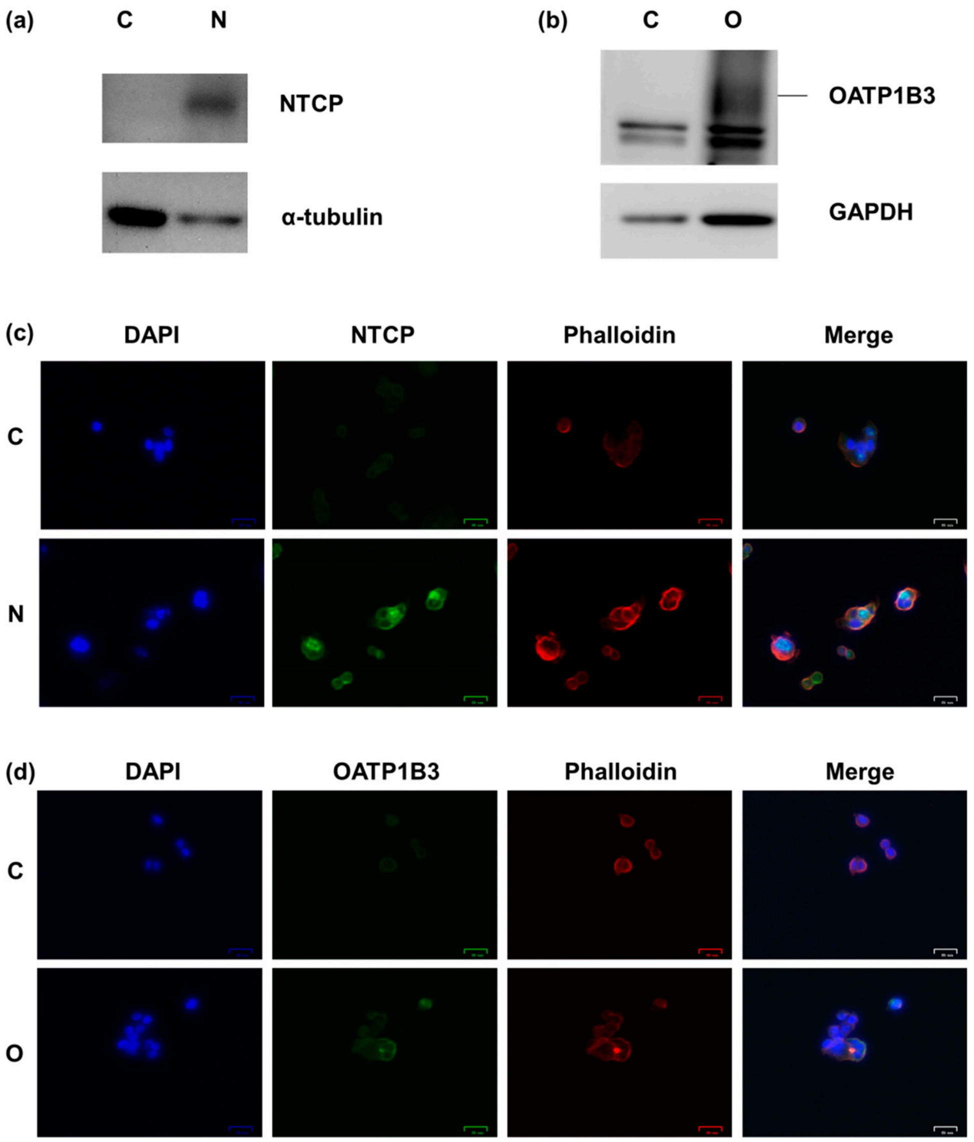

Figure S1. Genetic engineering of NTCP and OATP1B3. (a) And (b) Western blot analysis NTCP (38kDa) and internal control α-tubulin (50kDa) in control and *NTCP*-expressing HT-29. OATP1B3 (110kDa) and internal control GAPDH (37kDa) in control and *OATP1B3*-expressing HT-29. (c) And (d) Fluorescence microscopy showing the staining for NTCP (green) in (c), OATP1B3 (green) in (d), actin (phalloidin, red), and nuclei (blue). C: control HT-29. N: *NTCP*-expressing HT-29. O: *OATP1B3*-expressing HT-29.

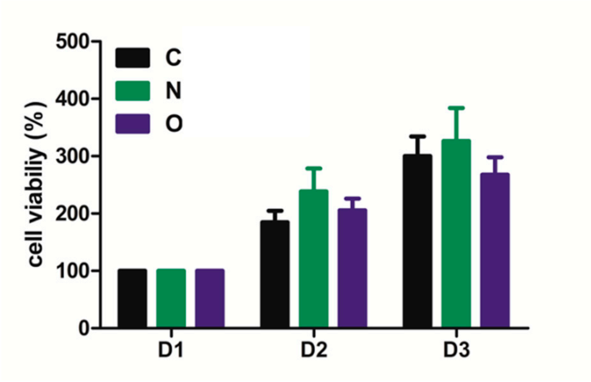

Figure S2. Proliferation ratio of NTCP and OATP1B3 overexpression. No significant differences were observed. Error bars represent the standard error of the mean. C: control HT-29. N: *NTCP*-expressing HT-29 cells. O: *OATP1B3*-expressing HT-29 cells. N = 4.

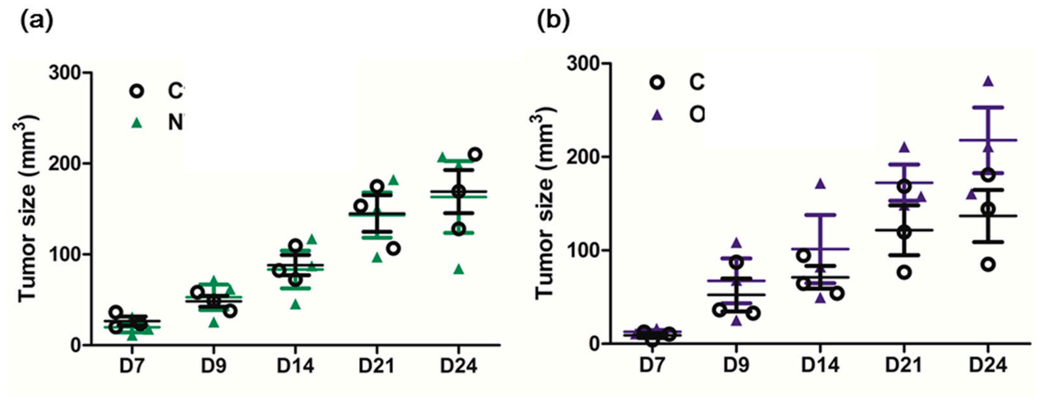

Figure S3. Tumor size. (a) *NTCP*-expressing tumor-bearing mice. (b) *OATP1B3*-expressing tumor-bearing mice. Tumor sizes were evaluated on day 7, 9, 14, 21, and 24 after xenograft. Error bars represent the standard error of the mean. C: control tumor. N: *NTCP*-expressing HT-29 cells. O: *OATP1B3*-expressing HT-29 cells.

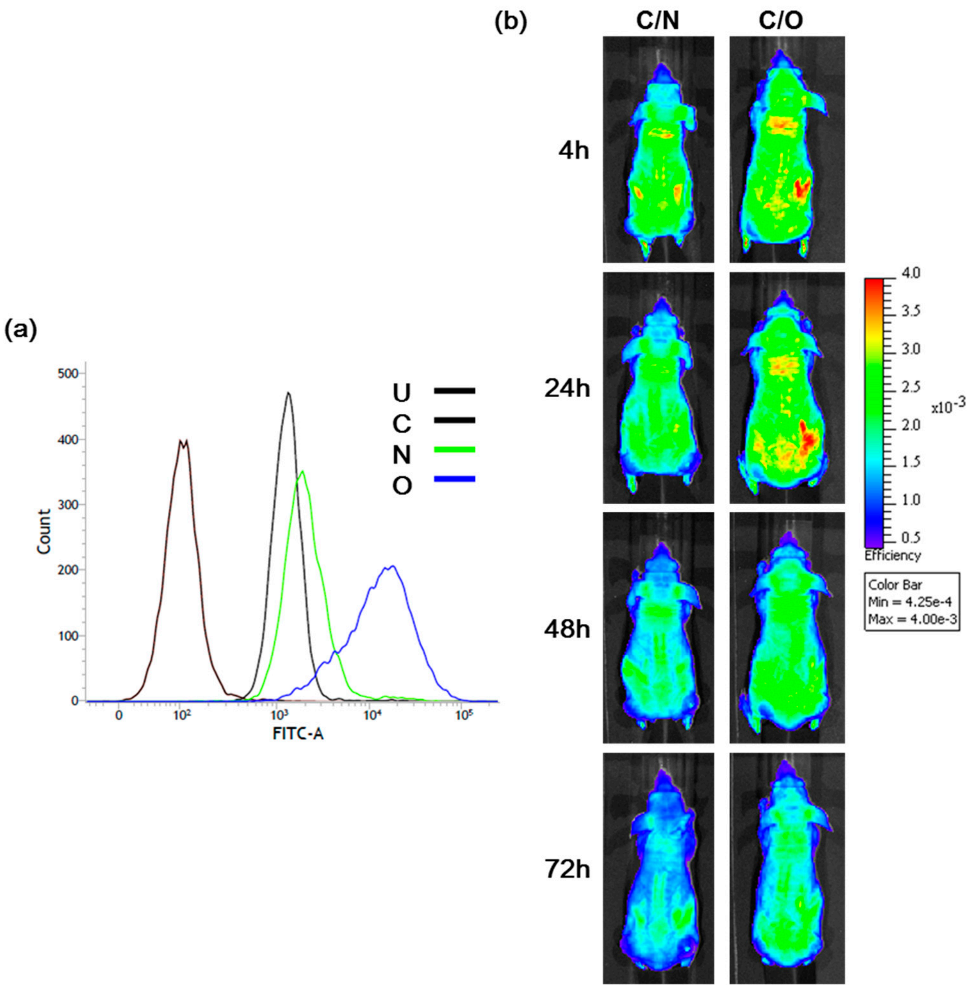

Figure S4. Assessment of FITC intake *in vivo* in the IVIS system. (a) Cells were measured using flow cytometry at the FITC wavelength to observe FITC intensity. (b) The ICG signal was observed using IVIS at 4, 24, 48, and 72 h after injecting ICG into *NTCP*- and *OATP1B3*-expressing tumor-bearing mice. The tumor on the left hind leg was the control tumor, and on the right was the *NTCP*-expressing tumor (top) and *OATP1B3*-expressing tumor (bottom). U: untreat. C: control HT-29 cells. N: *NTCP*-expressing HT-29 cells. O: *OATP1B3*-expressing HT-29 cells.
